# Reviews and Book Notices

**Published:** 1881-12

**Authors:** 


### Reviews and Book Notices.

ARTICLE XIII.—THE DISEASES OF CHILDREN. By WILLIAM HENRY DAY, M.D. Second Edition. 8vo., cloth, pp. 752; $5.00. Philadelphia, Presley Blakiston. Chicago: W. T. Keener, 96 Washington Street. 1881.

This is one of the best works on the diseases of childhood. It is complete, well written, entirely practical, embraces the latest views, and is based on the wide experience of Dr. Day in private and hospital practice. The chapters on Hygiene, Debility and Dentition are very well written, and will prove useful to every reader, notwithstanding the opinion of a few critics to the contrary. The chapter on typhoid fever in infants evinces the fact that the disease is quite common, and has just as often been overlooked by the majority of physicians in the past, being treated as infantile diarrhœa or remittent fever, and many of the ablest practitioners to-day will substantiate this statement.

The statement that simple diarrhœa may pass into all the other varieties is very sensible, and so is the author's treatment of those diseases. A variety of useful prescriptions, specially adapted for busy practitioners, are found through the volume, and by themselves at the end; which arrangement is very commendable. The two last chapters, devoted to diseases of the ear and diseases of the skin, although necessarily brief, will prove useful.

Chapter XVII, given to Intestinal Worms, begins thus: "The presence of worms in the intestinal canal is one of the commonest troubles of childhood." Although this statement is also made by some of the best writers of the continent, it does not hold good in this country, for one reason or another. However, this work is well suited for students and practitioners, although not any more so than some American works on the same subject. On the whole, the profession will be thankful to Dr. Day for this new edition of his valuable book. H.D.V.

---

ARTICLE XIV.—CYCLOPÆDIA OF THE PRACTICE OF MEDICINE. ZIEMSSEN. Vol. IX. Diseases of the Liver and Portal Vein, Interstitial Pneumonia. Pp. 928; Id. Supplement, pp. 844. William Wood & Co. 1880–1881.

These two volumes, together with the index, form the completion of perhaps the most important effort at unifying the science of medicine. It is a great credit to Germany to have thus gathered in a small compass all the medical genius of a people preëminent in all sciences. No writer on any disease would be pardoned to-day if he had not had some acquaintance with Ziemssen's Cyclopædia, yet for reasons which we will presently state, such cyclopædias present many disadvantages. We see one of these in this ninth volume, where the subject of Diseases of the Liver has been stretched out of proportion to all others to fill 836 pages, though otherwise well treated.

The supplement evinces another disadvantage, and several contributions are too deeply stamped with the "busy practitioner,"—resembling somewhat the usual "Society Transactions." However, the contributions to Syphilis, by Prof. J. N. Hyde, of Chicago; to Diseases of the Pleura, by G. M. Garland, M.D., of Boston; to Diseases of the Nose and of the Pharynx, by J. Solis-Cohen, M.D., and a few others, are as remarkable for their value as for their brevity.

The few illustrations accompanying some of the articles are very poor.

The comparative and well deserved success of this work warrants the prediction that for a decade to come, the profession will be flooded with cyclopædias. Indeed, there is the Cyclopædia of Chemistry, that of Surgery, and others in view. Whatever their intrinsic value, a second edition of these works is almost impossible, on the one hand; and on the other, science has never exhibited before such wonderful changes as in the last thirty years. In the end, individual efforts generally prove the best. H. D. V.

